# Evolutionary trends of As51 satellite DNA and its colocalization with 45S ribosomal DNA: a conserved feature in Characiformes fishes

**DOI:** 10.1186/s12864-026-12792-x

**Published:** 2026-04-01

**Authors:** Leticia Masiero Desajacomo, Rodrigo Zeni dos Santos, Duílio Mazzoni Zerbinato Andrade Silva, Marcelo Bello Cioffi, Gustavo Akira Toma, Fábio Porto-Foresti, Ricardo Utsunomia

**Affiliations:** 1 Departamento de Ciências Biológicas, Faculdade de Ciências, Universidade Estadual Paulista, UNESP, Campus de Bauru, Bauru, São Paulo, Brazil; 2 Cell and Developmental Biology Center, National Heart, Lung, and Blood Institute, National Institutes of Health, Bethesda, MD USA; 3 Departamento de Genética e Evolução, Universidade Federal de São Carlos, Campus de São Carlos, São Carlos, São Paulo, Brazil

**Keywords:** Intergenic spacer, Repetitive sequences, rDNA, Shared sequences, Tetra fish

## Abstract

**Background:**

Satellite DNAs (satDNAs) can arise from diverse genomic regions, but determining their precise origin remains a major challenge. A previous study reported the colocalization of the As51 satDNA and the 45S ribosomal DNA (rDNA) in *Astyanax janeiroensis*, suggesting a potential evolutionary link between these repetitive elements. However, this observation was based on a single observation and has not been broadly tested. In this study, we investigate whether the association between As51 satDNA and 45S rDNA extends to additional species within Characidae and Characiformes, aiming to uncover broader patterns that may provide insights into the evolutionary origin and dynamics of these genomic components.

**Results:**

Using multiple complementary datasets, including raw long-read and short-read sequencing, publicly available genome assemblies, and RNA-seq datasets, alongside customized bioinformatic approaches, we assembled and analyzed 45S rDNA arrays from several Characiformes species. We identified As51-like sequences within the intergenic spacers (IGS) of 45S rDNA across all examined species, with sequence similarity ranging from 45.10% to 90.20%. Notably, the tetra fishes *Astyanax mexicanus* and *Psalidodon paranae* exhibited exceptionally high abundance and sequence conservation of As51-like repeats within their rDNA spacers. Fluorescence in situ hybridization (FISH) validated the physical colocalization of As51 satDNA and rDNA clusters in these species.

**Conclusions:**

Our findings suggest that the As51 satellite DNA may have originated from the intergenic spacer region of 45S rDNA in an ancestral Characiformes species. The high sequence conservation and abundance observed in tetra fish indicate that this satellite DNA expanded within the spacer regions of certain Acestrorhamphidae and was subsequently dispersed into heterochromatic regions typically occupied by this satellite. This study provides new insights into the evolutionary dynamics of repetitive DNA sequences and offers a model for investigating how satellite DNAs can originate from functional genomic regions and spread across the genome.

**Supplementary Information:**

The online version contains supplementary material available at 10.1186/s12864-026-12792-x.

## Background

Satellite DNAs (satDNAs) are tandemly repeated sequences that constitute a substantial fraction of eukaryotic genomes and evolve through concerted evolution mechanisms involving unequal crossing-over and gene conversion [1–3]. These repetitive elements exhibit remarkable evolutionary plasticity, with rapid changes in sequence composition and copy number occurring across relatively short evolutionary timescales, while being predominantly localized within heterochromatic regions [4, 5].

Satellite DNAs originate through several molecular mechanisms that can produce tandemly repeated sequences from different genomic regions. Simple sequence repeats can arise through polymerase slippage during DNA replication, while longer satellite sequences may originate from rolling-circle replication events, unequal crossing-over between dispersed repetitive elements, or multiple insertions of transposable elements at the same genomic location [2, 5]. Once established, these tandem arrays can undergo rapid expansion or contraction through recombination-mediated mechanisms, with their persistence being favored in heterochromatic regions with reduced recombination rates.

Characiformes fishes (Teleostei, Otophysa) represent exceptional models for understanding satellite DNA evolution due to their remarkable cytogenetic diversity and species richness [6]. Within this order, the genera *Astyanax* and *Psalidodon* have emerged as particularly valuable systems for cytogenomic investigations, exhibiting complex phylogenetic relationships and extensive diversification of repetitive DNA sequences [7–9].

The As51 satellite DNA, first characterized in *Psalidodon scabripinnis* [10], has become one of the most well-studied chromosomal markers in Neotropical fishes. This AT-rich, 51-bp repetitive element has been extensively mapped across numerous *Astyanax* and *Psalidodon* species [11–14], with recent comparative genomic analyses revealing significant evolutionary diversification within this satellite family [9]. Two distinct derived variants have been identified in *Psalidodon*: a 39-bp variant shared by *P. bockmanni* and *P. paranae* (estimated divergence ~ 6.5 million years ago), and a 43-bp variant exclusive to *P. fasciatus* (~ 2 million years ago) [9].

Beyond this structural diversification, As51 has also been implicated in functional aspects of genome organization. Vicari et al. [15]. reported its colocalization with 18 S ribosomal DNA in *A. janeiroensis*, where it appeared associated with transcriptional silencing of ribosomal genes. The authors further suggested that As51 may share evolutionary relationships with transposable elements, potentially explaining its presence within ribosomal gene spacer regions.

The 45S ribosomal DNA units, organized within nucleolar organizing regions (NORs), contain highly conserved ribosomal genes (18 S, 5.8 S, 28 S) separated by rapidly evolving spacer regions that show considerable sequence variation among species [16, 17]. The intergenic spacer (IGS) regions are particularly dynamic, containing diverse repetitive elements that can influence ribosomal gene cluster structure and function [18].

In this study, we build upon previous findings showing the co-localization between As51 and 45S rDNA in *A. janeiroensis* and take advantage of the increasing availability of genomic resources for related species to investigate the evolutionary origin and genomic context of the As51 satellite DNA. We specifically asked whether (i) As51 is also associated with 45S rDNA across other *Astyanax* and *Psalidodon* species, and (ii) As51-like sequences occur in additional Characiform lineages.

## Results

### Assembly of the 45S rDNA region in the species shows the presence of As51-like satellite DNA within this region

Partial or complete 45S rDNA units were assembled in all analyzed species. The reconstructed arrays vary in length, and the data is summarized in Table 1. For taxa with long-read data and genome assemblies, a customized script enabled the reconstruction of tandem arrays encompassing complete 45S rDNA repeats. This approach provided a detailed characterization of the element, revealing both conserved organizational features and species-specific variations. Within these arrays, As51-like sequences were identified in the intergenic spacer regions, showing heterogeneous similarity levels and variable copy frequencies among species.

**Table 1:** 45S rDNA consensus length, genomic proportion, and As51-like annotations parameters on the assembly 45S rDNA in all species analyzed

	Species	45S rDNA consensus length	Genomic proportion	Number of As51-like annotations	Similarity with As51 satDNA
Genome and long-read sequencing	*Apareiodon* sp.	17,875 bp	-	112	50.94%–70.59%
	*Astyanax mexicanus*	10,537 bp	0,218%	1206	50.00%–92.16%
	*Hoplias malabaricus*	25,952 bp	0,591%	742	50.00%–62.07%
	*Ictalurus balsanus*	12,936 bp	0,107%	34	50.72%–60.38%
	*Pangasianodon hypophthalmus*	11,781 bp	0,273%	63	50.00%–60.78%
	*Piaractus mesopotamicus*	12,718 bp	0,105%	123	50.91%–64.71%
	*Psalidodon paranae*	21,890 bp	0,164%	95	50.98%–92.31%
Short-read sequencing	*Astyanax lacustris*	8,641 bp	0,445%	1	50.00%
	*Astyanax mexicanus*	9,155 bp	0,189%	5	54.90%–86.27%
	*Bario sanctaefilomenae*	10,769 bp	0,012%	5	50.91%–63.16%
	*Characidium gomesi*	10,811 bp	0,102%	5	65.38%–80.77%
	*Hoplias malabaricus*	11,212 bp	0,168%	2	50.00%–56.86%
	*Leporinus friderici*	8,605 bp	0,106%	6	53.57%–54.90%
	*Megaleporinus macrocephalus*	11,438 bp	0,149%	7	50.82%–77.36%
	*Prochilodus lineatus*	9,891 bp	0,260%	4	57.89%–62.75%
	*Psalidodon fasciatus*	9,588 bp	0,293%	4	56.60%–82.35%
	*Psalidodon schubarti*	8,998 bp	0,610%	4	57.41%–76.92%
	*Pygocentrus nattereri*	33,010 bp	0,179%	2	53.70%–54.39%
	*Serrapinnus notomelas*	10,413 bp	0,123%	10	50.00%–55.93%

Coverage analyses corroborated the accuracy of the 45S rDNA reconstructions through regional remapping, revealing uniform read depth across the entire 45S rDNA unit, although the proportion of mapped reads to the ribosomal cluster varied among species (Figure S1). The lowest coverage was observed in *Bario sanctaefilomenae* (≈ 0.012%), while *P. schubarti* showed the highest (≈ 0.610%). Despite the relatively low genomic proportion of *P. paranae* (0.164%), mapping intensity was notably higher in regions associated with the As51 satDNA, suggesting its substantial presence in the genome and supporting its role as the most abundant satellite DNA in the species’ genome. Summary statistics for the 45S rDNA consensus length, As51 satDNA characteristics, and genomic proportions derived from Bowtie2 remapping of both short- and long-read datasets are presented in Table 1.

Detailed inspection of the reconstructed arrays revealed distinct organizational patterns among taxa. Of the species analyzed, *P. paranae* was the only one for which long-read data were available. From this dataset, ten sequences were retrieved, encompassing five 45S rDNA clusters, all interspersed with As51 satellite DNA.

**Fig. 1:**
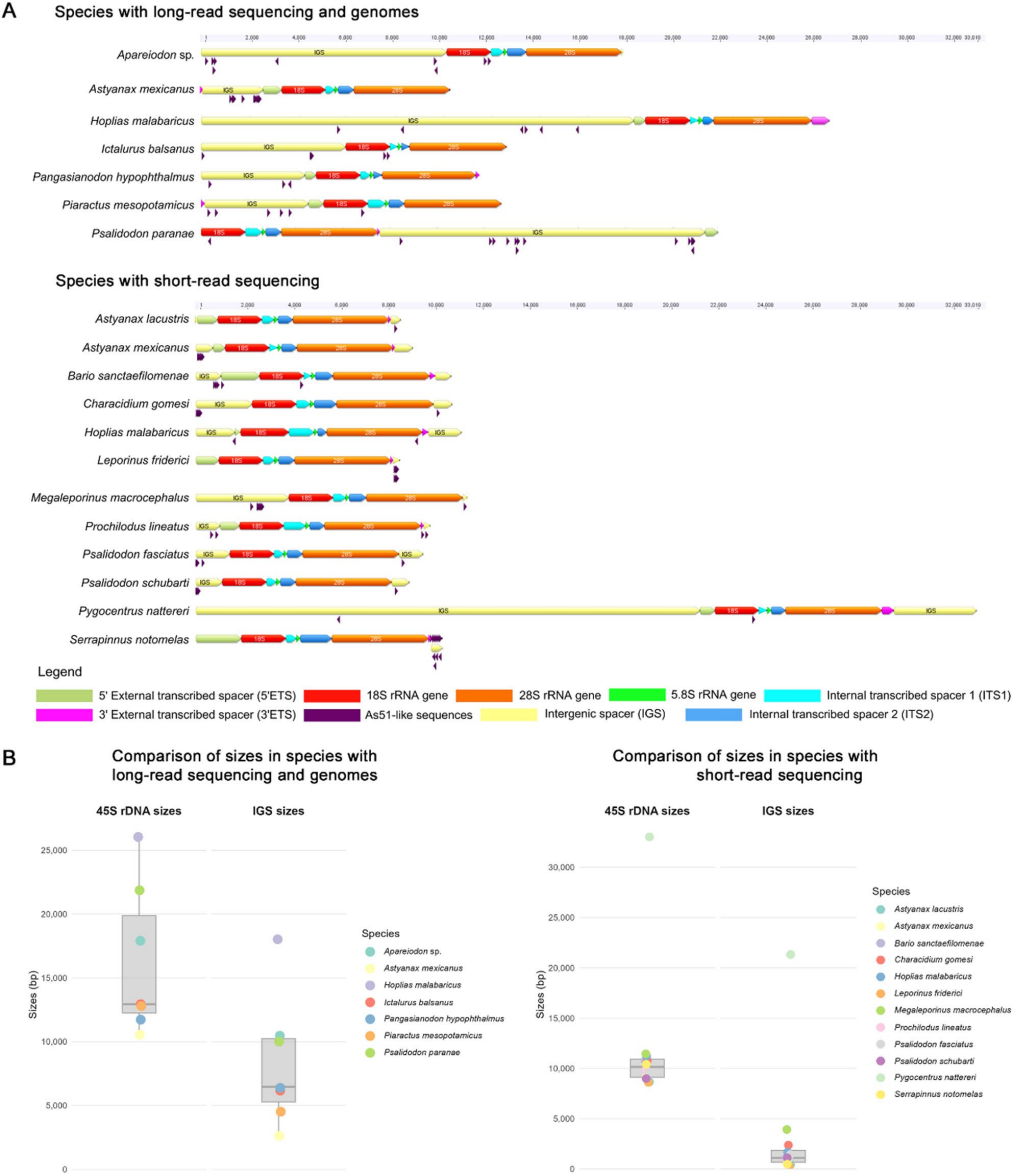
Comparative characterization of 45S rDNA loci across species. In (**A**), Assembled 45S rDNA units for each species, organized according to the genomic sequencing data type used for reconstruction; and in (**B**), Boxplot distributions of total 45S rDNA cluster lengths and intergenic spacer (IGS) sizes across species, stratified by sequencing data type

Among species with assembled genomes, *Apareiodon* sp. yielded two scaffolds containing ten tandemly repeated 45S rDNA units, in which a few As51-like sequences were identified. In contrast, the tetra *A. mexicanus* presented 33 sequences comprising eighty-five 45S rDNA units, most of them highly interspersed with As51 sequences. For *Hoplias malabaricus*, 65 sequences were recovered, encompassing 104 45S rDNA units, most clusters containing only a few As51-like sequences. *Piaractus mesopotamicus* presented fifteen sequences comprising fifty-four 45S rDNA units, while four sequences were extracted from the Siluriform *Ictalurus balsanus*, totaling twenty-five 45S rDNA clusters that contained only sparse As51-like fragments. Conversely, *Pangasianodon hypophthalmus* yielded two sequences with thirteen 45S rDNA clusters, densely embedded with As51 annotations (Fig. 1).

The MEGAHIT–SPAdes workflow was applied to assemble the 45S rDNA cluster in all species for which only short-read sequencing data were available. In most cases, the ribosomal region was reconstructed automatically, while in others it was necessary to merge separately annotated ribosomal genes to obtain the complete cluster. Owing to the k-mer–based assembly strategy and the limitations of short-read data, the resulting assemblies frequently lacked large portions of the intergenic spacers, which are enriched in repetitive sequences. Nevertheless, a few As51-like annotations were detected within these clusters despite the assembly challenges, as summarized in Table 1.

The length of the consensus 45S rDNA obtained from the MEGAHIT-SPAdes assembly demonstrated slight variation across Characiformes species, except for *Pygocentrus nattereri*. The similarity rate of the As51-like sequences was highly observed even in the earliest lineages to diverge within Characiformes, such as *Characidium gomesi*. The tetra species *A. mexicanus* and *P. paranae* exhibited much higher abundance of As51 satDNA than other species, with similarity rates reaching 92.16% and 92.31%, respectively. Notably, in *A. mexicanus*, the As51-like annotations were consistent across different sequencing data types, reinforcing the reliability of the distinct methodological approaches.

### Mapping with RNA-seq data revealed monomers of As51 satellite DNA in the intergenic spacer between 28 S and 18 S rRNA genes

Using available RNA-sequencing data, we mapped reads onto the coding region of the 45S rDNA cluster. Based on paired-end information and coverage patterns, we were able to manually delimit all subunits of the 45S rDNA array. As expected, the highest read coverage was observed in the 18 S, 5.8 S, and 28 S ribosomal RNA (rRNA) genes, whereas comparatively lower coverage was detected in the external transcribed spacers (Figure S2; ETS). This allowed us to define the boundaries of the non-transcribed intergenic spacer (IGS), within which As51 satDNA monomers were consistently identified between the 28 S and 18 S rRNA genes across all species (Fig. 1A). For species lacking RNA-seq data, the entire sequence flanking the rRNA genes was considered as the IGS.

The relationship between all species and the sizes of both the intergenic spacer (IGS) and the 45S rDNA array is presented in Fig. 1B. Among taxa with long-read or genome-based assemblies, *H. malabaricus* exhibited the longest 45S rDNA array and IGS, whereas the tetra *A. mexicanus* showed the shortest in both parameters. *P. paranae* displayed the highest number of As51-like sequences within the IGS. Notably, the three species with the longest IGSs (*Apareiodon* sp., *H. malabaricus*, and *P. paranae*) also presented the largest 45S rDNA arrays. Statistical analysis confirmed a highly significant positive correlation between IGS size and 45S rDNA consensus length (Spearman’s ρ = 0.937, *p* < 0.0001), providing strong evidence that IGS expansion may be the primary driver of variation in 45S rDNA unit length across Characiformes species.

In assemblies based on short-read sequencing, the lack of continuous tandem repeats precluded direct comparisons of IGS lengths among species. Nevertheless, As51-like fragments were consistently identified within these regions, preserving the characteristic tandemly repeated organization. In *A. mexicanus*, the detection of As51-like sequences in the consensus 45S assembly aligned with the pattern observed in the genome-based reconstruction, thereby confirming the reliability of both assembly approaches. Additionally, *P. nattereri* exhibited the most extensive 45S rDNA array and IGS among the short-read assemblies, showing marked divergence in cluster size relative to the other species.

### Notable similarity between the conserved As51 satDNA and the As51-like sequences annotated at the intergenic spacer

We selected the array used for the assembly of the 45S rDNA cluster. Array size and the number of 45S tandem repeats are summarized in Table 2. Subsequently, we extracted all the As51-like sequences presented in the array and verified a notable similarity between these sequences and the consensus As51 satDNA, considering the different groups and the distance among them (see the alignments in Figure S3). Detailed information is also presented in Table 2.

**Table 2:** Data on the array, 45S tandem repeats and Muscle alignment results on the species with assembled genomes. The percentages are based only on the unique monomers

Species	Size of the array	45S tandem repeats quantities	Total of monomers retrieved	Unique monomers retrieved	Pairwise identity with As51 satDNA	Similarity range of the monomers with As51 satDNA
*Apareiodon* sp.	248,379 bp	11	118	13	69.20%	54.90%–70.59%
*Astyanax mexicanus*	128,904 bp	11	112	11	79.50%	54.90%–92.16%
*Hoplias malabaricus*	16,237 bp	1	14	7	53.60%	50.00%–55.17%
*Ictalurus balsanus*	194,934 bp	10	10	1	53.13%	53.13%
*Pangasianodon hypophthalmus*	261,731 bp	14	57	12	43.10%	50.00%–60.78%
*Piaractus mesopotamicus*	210,038 bp	8	18	8	54.70%	50.94%–64.71%
*Psalidodon paranae*	37,791 bp	2	28	28	73.20%	58.82%–90.38%

**Fig. 2:**
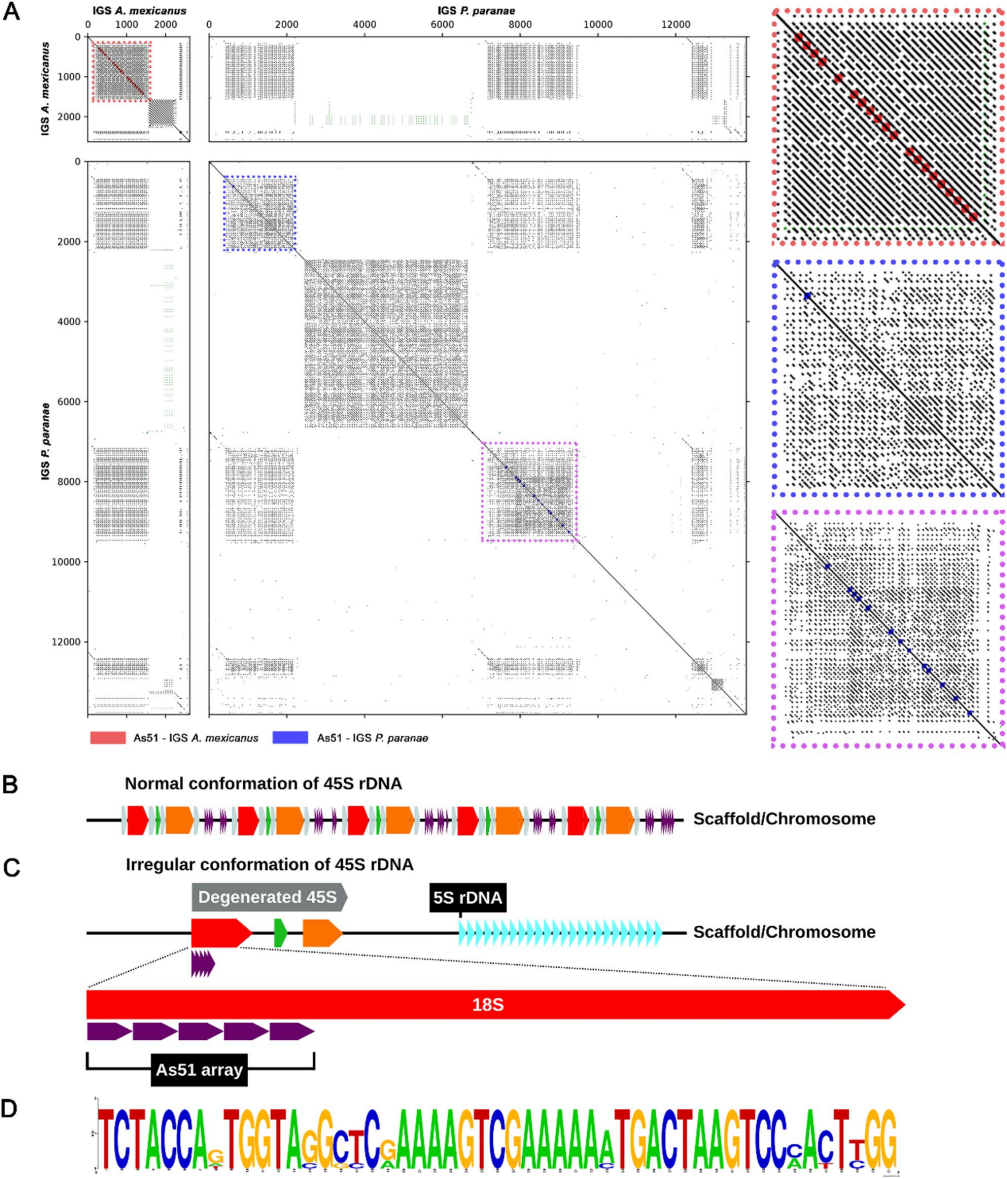
As51 satDNA pattern in tetra fish. In (**A**), a comparative dotplot of *Astyanax mexicanus* and *Psalidodon paranae*’s intergenic spacer (IGS), with the highlight of the satDNA repeated monomers (in red, the satDNA in *A. mexicanus*, and in blue, the satDNA in *P. paranae*); in (**B**), normal conformation of the region embedded with 45S rDNA; in (**C**), the degenerated 45S found in *Astyanax mexicanus* with the presence of an As51 array, commonly found with major 5 S rDNA clusters; and, in (**D**), the sequence logo resultant of the alignment of all monomers of As51 found within the 18 S of *A. mexicanus*

Representatives of the Acestrorhamphidae family exhibited an alignment with slight base modifications in the As51 satDNA sequence compared with the other groups, with a high overall similarity between the sequences extracted from the arrays and the consensus As51 satDNA. The similarity among As51-like monomers reached approximately 90%, indicating a close relationship with the conserved As51 satDNA found in tetra species. Given the strong similarity to the As51 consensus and their phylogenetic proximity, we compared the IGS regions of *A. mexicanus* and *P. paranae* to investigate potential structural patterns (Fig. 2A).

The comparative dotplot revealed the highly repetitive nature of these spacers, characterized by large blocks and subrepeats. In *A. mexicanus*, As51-like sequences occupy about 1,300 bp of the IGS, forming short tandem arrays of a few monomers, whereas in *P. paranae* they extend over approximately 1,500 bp, also organized in small tandem clusters. Despite differences in size and sequence composition, the dotplot highlights a clear structural similarity between the two species.

To investigate the genomic distribution and potential association of the As51 satellite DNA with the 45S rDNA, we performed BLASTn searches against the *A. mexicanus* reference genome. As51 arrays were confined to 45S rDNA regions, with a distinct array embedded in a degenerated 18 S rRNA gene (Fig. 2C). Degenerated 45S rDNA units containing truncations in the 28 S rRNA gene were identified as a single-copy locus on one chromosome and as dispersed copies across seven genome scaffolds. Interestingly, they frequently occurred in close association with major 5 S rDNA clusters (Fig. 2C).

Moreover, these degenerated 45S rDNA units exhibited substantial divergence in both the internal and external transcribed spacer regions, as well as within the 18 S rRNA sequence containing the As51 insertion, when compared with species-specific 18 S rRNA references available in NCBI (Figure S4A). This finding suggests that As51 integration into the 45S rDNA may contribute to structural variation and sequence divergence within rDNA arrays in *A. mexicanus*. In this case, the As51 arrays consisted of 4–6 monomers, typically arranged in tandem near the 5′ end of the 18 S rRNA gene, spanning up to 430 bp. Two identical degenerated 45S rDNA units were identified on separate scaffolds (Figure S4B). Analysis of the 40 As51 monomers embedded within the 18 S rRNA gene revealed sequence identities ranging from 88.24% to 92.16% relative to the consensus As51 sequence, which could be grouped into six distinct variants. Alignment of all monomers against the consensus sequence yielded a mean pairwise identity of 93.20%, with the corresponding sequence logo shown in Fig. 2D.

### Fluorescence in situ hybridization in tetra species demonstrates the colocalization of As51 and the ribosomal genes

**Fig. 3:**
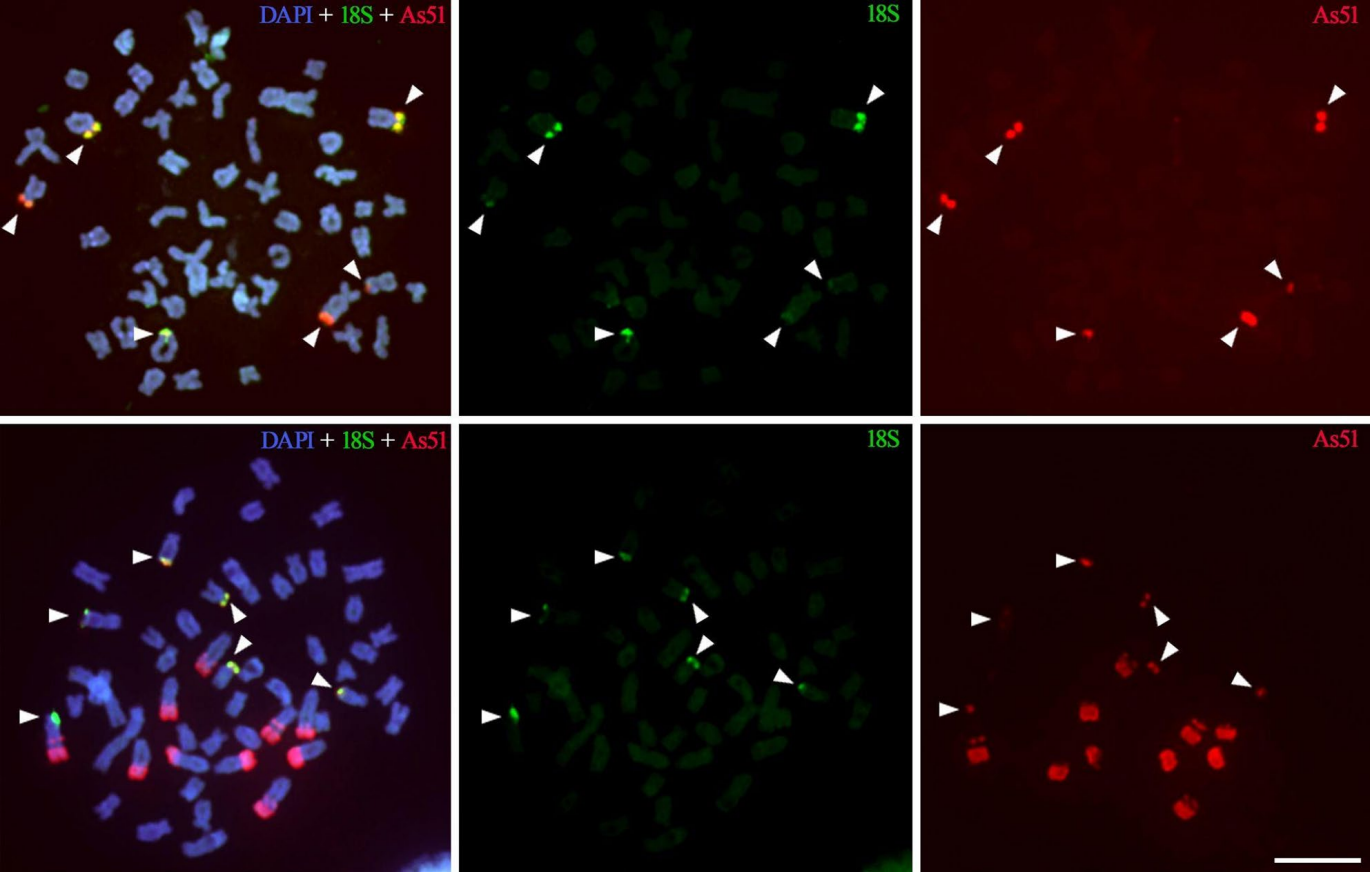
Mitotic metaphases of *Astyanax lacustris* (above) and *Psalidodon paranae* (below) after double-FISH with 18 S rDNA (green) and As51 satDNA (red). Arrowheads indicate colocalized 18 S rDNA and As51 signals. Note also the presence of large As51 blocks without any detectable 45S rDNA signals in heterochromatic regions of acrocentric chromosomes in *Psalidodon paranae*. Bar = 10 μm

Double-FISH experiments using As51 and 18 S rDNA probes corroborated our in silico findings. In *Astyanax lacustris*, all As51 clusters were found colocalized with 18 S rDNA sites. In contrast, in *P. paranae*, although all 18 S rDNA clusters showed colocalization with As51, large independent arrays of As51 were also detected in the long arms of subtelocentric and acrocentric chromosomes, without any evidence of nearby rDNA sequences (Fig. 3).

## Discussion

Genome skimming and advances in bioinformatic pipelines have greatly improved our understanding of tandemly repeated sequences, including satellite DNAs and multigene families, as well as their genomic distribution patterns [5, 19]. These approaches are particularly effective for shorter tandem repeats (< 1000 bp), which can be reliably reconstructed from low-coverage sequencing data. While 5 S rDNA has been widely studied due to its short and uniform repeat units [20], 45S rDNA, with repeats spanning tens of thousands of nucleotides, remains challenging to analyze because of its length, complex secondary structures, and assembly difficulties. Recent advances in specialized bioinformatic pipelines and long-read sequencing have improved the reconstruction of large satellite DNAs and near-complete rDNA arrays, such as 45S repeats [21–23]. In this study, we combined low-coverage short-read data for some species with long-read data and complete genome assemblies for others, along with RNA-seq data and double-FISH experiments, applying pipelines tailored to repetitive DNA to reconstruct and characterize both the coding regions and adjacent intergenic spacers of 45S rDNA.

Our approach successfully reconstructed the 45S rDNA units across multiple Characiformes species, achieving complete assemblies in some cases, revealing both conserved structural features and species-specific variations in repeat organization. The canonical gene order 18 S-ITS1-5.8 S-ITS2-28 S was consistently maintained across all investigated taxa, confirming the fundamental conservation of ribosomal gene arrangement in vertebrates (Fig. 1A). However, a substantial variation was observed in the overall length of complete rDNA units, ranging from approximately 10 kb in the most compact arrangements (i.e., *A. mexicanus*) to over 33 kb in species with extensively expanded intergenic spacers (i.e., *P. nattereri*). The sizes of IGS widely varied among species (median = 2,380 bp; range = 295–21,311 bp), indicating that differences in rDNA unit length may be largely driven by IGS expansion (Fig. 1B). IGS sequences exhibited limited conservation among distantly related species. Nevertheless, a shared feature across taxa is the pervasive presence of subrepeats, frequently organized in tandem, which likely contribute to structural variability within the IGS (Fig. 2A; Figure S5–S6).

The results presented here also provided molecular and cytogenetic evidence for a close evolutionary relationship between As51 satellite DNA and repetitive sequences residing in the intergenic spacer (IGS) of the 45S rDNA in Characiformes. The detection of As51-like sequences across all investigated Characoidei species, with similarity ranging from 45.10% to 90.20%, indicates a conserved phylogenetic signature. The occurrence of related motifs in multiple families, including Characidae and Acestrorhamphidae, as well as in representatives of Siluriformes, suggests that similar repetitive elements are broadly distributed among these lineages.

While this distribution is consistent with the possibility that related As51-like motifs were present early in the diversification of these groups, the present data do not allow us to determine whether this pattern reflects shared ancestry or recurrent amplification of similar motifs. Based on the observed sequence similarity and structural association with the 45S rDNA, we consider an evolutionary connection between IGS-associated repeats and As51 satellite DNA to be the most parsimonious interpretation.

The physical colocalization between As51 satDNA blocks and 45S rDNA clusters in *P. paranae* and *A. lacustris* provides cytogenetic support for the genomic association inferred from sequence-based analyses. While this pattern is unlikely to represent a purely coincidental association, it is more appropriately interpreted as being consistent with an evolutionary link related to the origin and subsequent dynamics of this satellite DNA. In most species analyzed, As51-like sequences were frequently found arranged in tandem within the intergenic spacer (IGS) of the 45S rDNA (Fig. 1). Taken together, these observations support the hypothesis that this association may reflect an evolutionary relationship established early in the diversification of Characiformes. However, alternative scenarios cannot be excluded, and broader comparative genomic analyses with expanded taxonomic sampling will be necessary to clarify the direction and timing of these events.

Ribosomal intergenic spacers are known for their rapid evolutionary dynamics, low selective constraints, and inherently repetitive structure composed of subrepeat arrays [24–26]. Such features create a favorable genomic environment for the generation of new sequences through mechanisms like replication slippage [27]. Once originated, these sequences can be released from the rDNA locus and subsequently amplified in other genomic regions [4]. Similar cases, in which IGS-derived sequences have given rise to new satellite DNA families, have been reported across diverse eukaryotic groups, including plants such as tomato [28, 29] and fish lineages [30]. Together, these observations suggest that the emergence of satDNAs from IGS regions may represent a recurrent process in genome evolution.

Our findings complement, but also provide a counterpoint to the hypothesis proposed by Vicari [15], who suggested that the colocalization between As51 and 45S rDNA in *Astyanax janeiroensis* could reflect a repressive interaction leading to ribosomal gene inactivation. While this interpretation may be valid for *A. janeiroensis*, our analyses extend the picture by showing that the colocalization of As51 satDNA and 45S rDNA is not restricted to this species and occurs in two other taxa analyzed here. Indeed, all ribosomal *loci* analyzed in our study contained As51-like sequences within the IGS.

Taken together, this evidence suggests that the case of *A. janeiroensis* may represent a lineage-specific exception, possibly associated with an unusual local expansion of As51 arrays in particular ribosomal *loci*. Such expansions could, in principle, reach a threshold sufficient to interfere with ribosomal gene function and promote NOR inactivation. Importantly, these observations indicate that it is likely not the mere presence of satellite DNA, but rather its local abundance, organization, and amplification dynamics that shape the functional outcomes of rDNA clusters. In this context, the detection of small tandem arrays of As51-like repeats within the 18 S rRNA gene region in *Astyanax mexicanus* may represent a potential mechanistic substrate for NOR instability or inactivation (Fig. 2C). Thus, interactions between satellite DNA and rDNA may range from largely neutral to potentially disruptive, depending on the extent and genomic context of satellite DNA accumulation.

The transition from a low-copy repetitive motif derived from the IGS to a high-abundance satellite DNA, such as As51 in several *Psalidodon* species, is a process involving multiple steps and genomic dynamics mechanisms, including the duplication of short IGS-derived motifs through replication slippage, followed by the progressive amplification of tandem arrays via unequal crossing-over [31]. These processes within the heterochromatic environment resulted in long tandem arrays spanning millions of nucleotides, explaining why As51 is the most abundant satDNA in several *Psalidodon* species [8, 9].

**Fig. 4:**
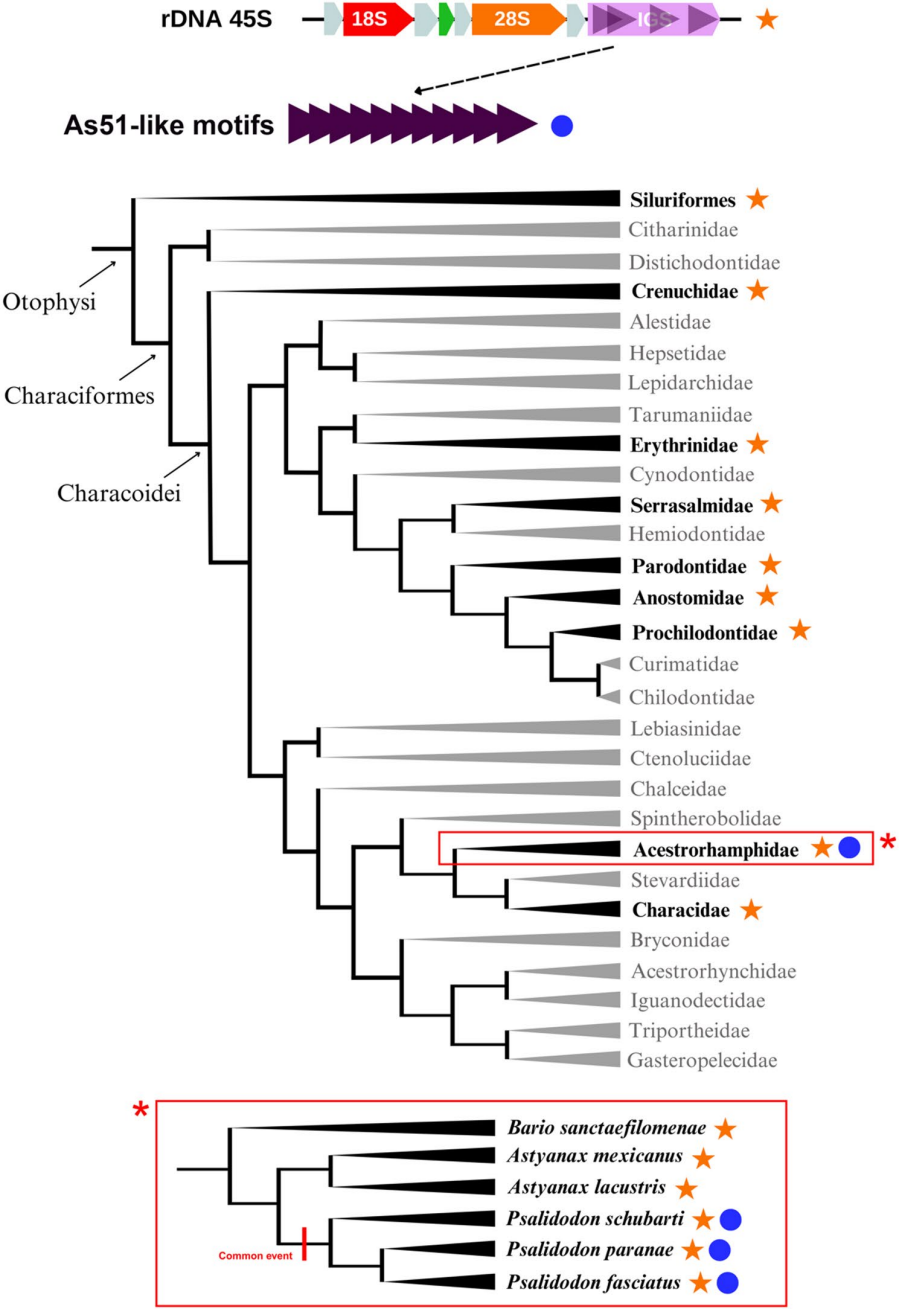
Evolutionary path of the As51 satellite DNA in Characiformes fish phylogeny and its spread in Acestrorhamphidae family. Phylogeny based on Oliveira [34] and Melo [35]

Particularly notable is the high similarity (80–90.20%) and abundance of these sequences in *A*. *mexicanus* and *P*. *paranae*, indicating a closer evolutionary relationship between As51 and the IGS in these species. This pattern of distribution and similarity supports the hypothesis that As51 satellite DNA may have originated from repetitive elements within the IGS of 45S rDNA in a common ancestor, followed by amplification and dispersion throughout the genome of some tetra species. This model of satellite DNA “birth” from pre-existing repetitive regions has been proposed in other organisms [3, 4, 32, 33], and our study provides more detailed evidence of this phenomenon in Characiformes fish (Fig. 4).

The evolution of satellite DNA is driven by population-level dynamics and copy number variation, resulting in a common feature of intraspecific variability [3–5]. Therefore, it is imperative to consider the variations presented in each taxa while addressing such elements. Despite the substantial size of the dataset utilized in this study, it encompasses a single data point and misses the intraspecific variation that is typically observed in satDNAs analyses.

The dynamics of As51-like sequences and their intraspecific variability, especially in related groups of Acestrorhamphidae in which As51 is a bona fide satDNA, have the potential to explore and evaluate if the expansion of these sequences is ongoing, episodic or stabilized intra-species. The As51 satellite DNA is highly dynamic within *Astyanax* and *Psalidodon* genera, whereas several cytogenetic studies have discussed its diversification in populations, rather this variability occurs in chromosome-level with spanning clusters [8, 10–13, 36] or in the nucleotide-level with multiple variants [9]. The analysis of copy number, sequence divergence and chromosomal distribution are important traits to overlook in population-level studies to infer the evolutionary of these motifs in IGS and could be potentially used to discuss if these sequences are actively evolving within the 45S rDNA. While we can trace an evolutionary path of these motifs in Characoidei, there are limitations caused by the lack of assessment of intra-species variation and dynamics.

## Conclusion

Our findings strongly suggest that the As51 satellite DNA, commonly used as a chromosomal marker in *Psalidodon* species, is a sequence belonging to the intergenic spacer region of the 45S rDNA cluster. The As51 sequence is speculated to have originated from this spacer in an ancestral Characiformes species, or perhaps previously in Otophysi, since this sequence was consistently found across all analyzed taxa from Characiformes and in Siluriform species as well, demonstrating the highly conserved characteristic assumed by this sequence. Thereby, the colocalization between As51 satDNA and 45S rDNA, observed in silico and in situ, provides substantial evidence that this association reveals an evolutionary vestige of the satellite’s origin.

The rapid evolutionary dynamics of the ribosomal intergenic spacer likely facilitated its expansion in Acestrorhamphidae. Subsequently, these sequences dispersed into heterochromatic regions typically occupied by this satellite in the *Psalidodon* genera. The results of this study support the role of IGS regions in the origin and emergence of satDNAs, highlighting the fundamental role of IGS-derived repeats in genome evolution and offering a model for investigating how satellite DNAs can originate from functional genomic regions and subsequently spread across the genome.

## Materials and methods

### Genomic data

To investigate the organization of 45S rDNA arrays and their relationship with As51 satellite DNA across Characiformes, we employed an integrative strategy based on different types of molecular data, including complete genomes, long-read sequencing, short-read sequencing, and RNA-seq data. A portion of these datasets was retrieved from the GenBank Nucleotide Database, while others were newly generated in the present study. Detailed accession numbers and metadata are provided in Supplementary Material Table S1.

Complete genome assemblies were obtained from public databases for the following species: *Astyanax mexicanus*, *Hoplias malabaricus*, *Apareiodon* sp., *Ictalurus balsanus*, *Pangasianodon hypophthalmus* and *Piaractus mesopotamicus*. These assemblies allowed for the reconstruction of full or nearly complete 45S rDNA units and precise mapping of As51-like sequences within or adjacent to rDNA clusters. Long-read sequencing data (PacBio) for *Psalidodon paranae* were obtained from the NCBI database, enabling the recovery of extended rDNA arrays as well.

Short-read sequencing data were analyzed for twelve species: *Astyanax lacustris*, *Astyanax mexicanus*, *Bario sanctaefilomenae*, *Characidium gomesi*, *Hoplias malabaricus*, *Leporinus friderici*, *Megaleporinus macrocephalus*, *Prochilodus lineatus*, *Psalidodon fasciatus*, *Psalidodon schubarti*, *Pygocentrus nattereri* and *Serrapinnus notomelas* (details in Table S1).

RNA-seq data, when available from public databases, were used to support the identification and delimitation of the transcribed portions of the 45S rDNA unit (18 S, 5.8 S, and 28 S), as well as the internal and intergenic spacers. Transcriptomes were analyzed for the following species: *A. lacustris*, *A. mexicanus*, *B. sanctaefilomenae*, *P. lineatus*, *P. paranae*, *P. nattereri* and *S. notomelas*.

**Fig. 5:**
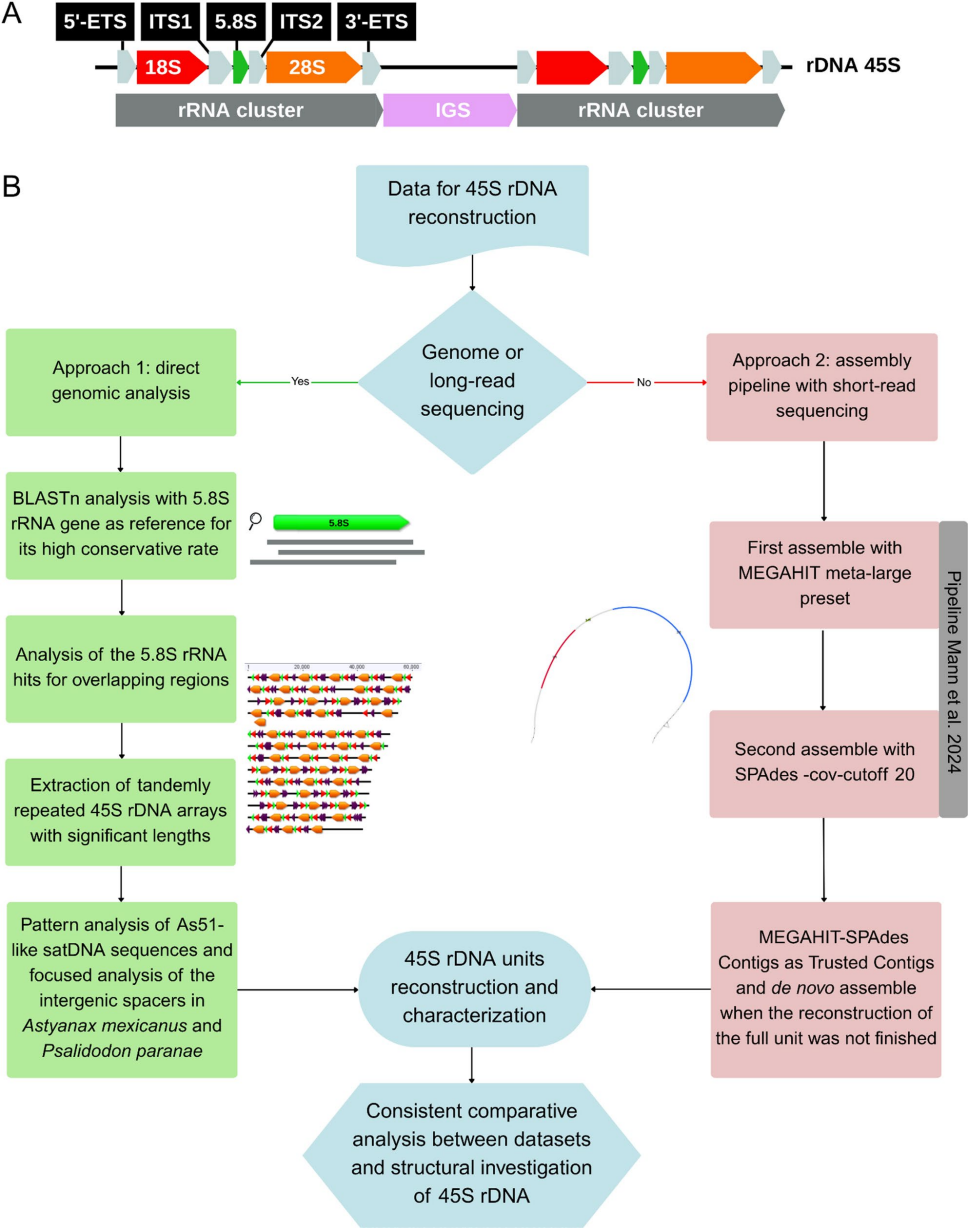
Methodological approach to the reconstruction of 45S rDNA. In **A**, a schematic representation of 45S ribosomal DNA sequence, showing the organization of external transcribed spacers (ETS-grey arrow), Internal transcribed spacers (ITS - grey arrow), rRNA clusters of gene families: 5.8 S (green arrow), 18 S (red arrow) and 28 S (orange arrow), and intergenic spacer region (pink arrow); and in **B**, the assembly’s workflow based on the type of data for each species

Depending on the type and quality of data available for each species, different strategies were applied to recover and analyze the 45S rDNA units, ensuring the most reliable reconstruction of the arrays for downstream comparative and evolutionary analyses (Fig. 5). The specific criteria for the As51-like classification were: similarity cutoffs at 50.00%; manually inspection of these sequences location; organization following structural patterns or a tandem repeat characteristic; manually evaluation of motifs and conservative patterns, specially in the alignments generated by Geneious; and length of the sequence at an average of 50–60 bp, but not as an excluding parameter.

### Assembly of 45S rDNA unit - complete genomes and raw long reads

For species with complete genomes and/or raw long-read sequencing available, we used a custom script with integrated BLASTn (Basic Local Alignment Search Tool) [37] to characterize the 45S rDNA arrays, with the 5.8 S rRNA gene serving as the reference due to its high conservation across taxa. This approach allowed for a standardized search across different fish groups within the order Characiformes and among external species within the order Siluriformes. BLASTn searches were performed against designated databases, and matches to the 5.8 S rRNA reference were analyzed for overlapping regions. When an overlap was detected, the script extended this region to recover tandemly repeated 45S rDNA arrays located within the same region, and when no further overlaps were identified, the sequences were then printed and extracted to the output file. This workflow permitted the extraction of matched sequences from the same region with more significant lengths, up until 326,000 bp, thereby enabling the investigation of the arrangement and distribution patterns of As51-like satellite DNA within the rDNA clusters of the analyzed species.

For data extraction, BLASTn output fields, including subject sequence identifier (sseqid), subject start position (sstart), and subject end position (send), were used to guide subsequent analyses. To further investigate As51-like sequences specifically within the intergenic spacer (IGS) regions, focused analyses were conducted on *A. mexicanus* and *P. paranae*, where extensive monomers of As51-like sequences were identified.

Scripts and resources used in this study are available at https://github.com/leticiamasiero/rDNA_blastn/tree/main.

### Assembly of 45S rDNA unit - raw short reads

When only raw short-read sequencing data were available, we applied the pipeline described in Mann [22] to assemble comprehensive consensus sequences from highly abundant repeats and explore their structural features. Briefly, after quality control and trimming, genome-skimming reads (short reads) were assembled in a first round using MEGAHIT (meta-large preset), followed by a second assembly round with SPAdes (--cov-cutoff 20), using the final MEGAHIT contigs as trusted contigs (https://github.com/crimBubble/repeats_and_circles_assembly). This approach allowed the recovery of representative 45S rDNA units even in the absence of long-read or genome assembly data, ensuring consistency across datasets for comparative analyses.

### Coverage analysis with Bowtie2

Utilizing Bowtie2 [38] and the samtools software, the 45S rDNA units retrieved from short-read or long-read sequencing and genomes were subjected to an alignment process to assess the coverage of the assembled unit against our libraries. For species with long-read sequencing or genome, two complete units of 45S rDNA were employed for this analysis. Conversely, for species with short-read sequencing, this analysis was performed with the unit assembled by MEGAHIT-SPAdes. In both cases, the following parameters were utilized: --sensitive, --no-discordant, --no-unal, and --no-mixed. Only *Apareiodon* sp. was excluded from the coverage analysis due to the absence of short reads deposited in the NCBI database. We used the maximum number of reads available in our libraries, with a range of 2 × 1,117,552 to 2 × 310,409,303 paired-end reads. The coverage analysis was then visualized using Geneious Prime software to ascertain the veracity of the assembled ribosomal regions.

### Annotation of 45S rDNA

When available (Table S1), we mapped species-specific transcriptome reads to the assembled 45S rDNA units to determine the transcription start site of the 18S rRNA gene, corresponding to the beginning of the 5’ external transcribed spacer (5’ ETS). This approach also allowed us to define the boundaries of the 3’ external transcribed spacer (3’ ETS) and the intergenic spacer (IGS). Mapping was performed using Bowtie2 [38] against two complete units of 45S rDNA in the *A. mexicanus*, *P. paranae*, *P. mesopotamicus* and *P. hypophthalmus* species and against one complete unit in *S. notomelas*, *P. lineatus*, *B. sanctaefilomenae*, *A. lacustris*, *P. nattereri*, *L. friderici* and *H. malabaricus*, with the following parameters: --sensitive, --no-discordant, --no-unal, and --no-mixed. The resulting alignments were visualized and manually inspected using Geneious Prime to confirm the precise boundaries of these regions.

### Muscle algorithm alignment of As51-like sequences

To treat a massive number of analogous sequences, the Muscle algorithm [39] aligner included within the Geneious software suite was employed. Due to its capacity to process extensive sequence data, Muscle was utilized to align and compare the As51-like sequences with the consensus As51 satDNA sequence obtained from the GenBank Nucleotide Database to investigate the similarity of this sequence. To accomplish this, the entire array used to characterize the 45S rDNA in species with long reads and genomes was selected and all As51-like sequences were manually extracted from the scaffold. The parameters were set to default, with 8 as the maximum number of iterations. Subsequent to the alignment of these As51-like sequences and the original As51 satDNA, we could verify the similarity rate and pairwise identity, in addition to the number of unique sequences found in each species.

### Statistical analysis of IGS and 45S rDNA array size relationship

To investigate the relationship between the intergenic spacer (IGS) size and the total 45S rDNA array length, we performed correlation analyses using data from all species (*n* = 19). Prior to correlation analysis, normality of both variables was assessed using the Shapiro-Wilk test. Based on the normality test results, Spearman’s rank correlation coefficient (ρ) was calculated to evaluate the strength and significance of the association between IGS size and 45S rDNA array length. All statistical analyses were performed using Python 3.11 with the SciPy library (version 1.16.3), and significance was set at α = 0.05.

### Flexidot for building self-dotplots and comparative dotplots

To visualize the repetitive features of intergenic spacers and highlight the As51-like sequences within them, Flexidot software [40] was used to create self-dotplots. When the intergenic spacer (IGS) was clearly delimited in long-read or genomic data, or in species with available RNA-Seq, dotplots were constructed using only this region. In species where the IGS could not be identified as a distinct component of the 3’- and 5’ETS, a unique spacer combining all three external spacers was used instead.

An alignment with BLASTn was performed to identify As51 satDNA hits within each species spacer using the parameters -max_target_seqs 100,000, -word_size 10, -evalue 1e-10, and -qcov_hsp_perc 60. These repeat hits were then used to highlight the dotplot. When no hits were found with the original As51 satDNA sequence, the As51-like sequence species-specific was used as the basis for the BLASTn alignment. The self-dotplot was built according to the software instructions, with the parameters -k 10, -S 1, -T 30, -E 15, -A 1, -m 0, and -P 20. The comparative dotplot construction between *A. mexicanus* and *P. paranae* utilized the following modificative parameters -m 2, -x, -k 10, -F 0.06, and -A 1.

### BLASTn in *Astyanax mexicanus*’s genome to investigate the relation between As51 satDNA arrays and the 18 S rRNA gene

To investigate the As51 satDNA pattern in the genome of tetra fish *A. mexicanus* and to evaluate if this satDNA presented monomers in tandem not related to the 45S rDNA region, we performed a BLASTn analysis both of the satDNA and the 45S rDNA against the genome in this species. We used -evalue 1e-10 and -qcov_hsp_perc 60 as parameters to the nucleotide alignment. The output data was subsequently converted into a .bed extension, wherein bedtools intersect (-v) was implemented to generate a unique sequence file of these hits, where each scaffold and/or chromosome were then extracted with samtools faidx to visualize its organization in Geneious Prime software.

### Fluorescence in situ hybridization (FISH)

We had available mitotic metaphase chromosome preparations of *A. lacustris* and *P. paranae*. Thus, to validate the results obtained in silico, we performed fluorescence in situ hybridization (FISH) experiments to assess the colocalization of As51 satDNA and rRNA genes. Double-FISH was carried out using 18 S rRNA and As51 satDNA probes. The As51 probe was directly labeled at the 5′ end with digoxigenin during its synthesis. In parallel, an 18 S rRNA probe from *H. malabaricus* [41] was labeled with Atto-488-dUTP using a nick translation kit (Jena Bioscience), following the manufacturer’s instructions. FISH experiments were performed according to Sassi [42]. We treated chromosome slides with RNAse (10 µg/mL in 2× SSC solution) for 1 h 30 min at 37 °C followed by pepsin treatment (50 µg/mL in 10 mM HCl) for 10 min. 70% formamide was used to denaturate the chromosomes at 72 °C for 3 min and 15 s. Hybridization was carried out by applying 20 µL of a mixture containing 200 ng of labeled probe, 50% formamide, 2× SSC, 10% SDS, 10% dextran sulfate, and Denhardt’s buffer (pH 7.0), and incubating the slides in a moist chamber at 37 °C for at least 14 h. For digoxigenin-labeled probes, signals were detected using an Anti-digoxigenin-rhodamine kit (Roche). Finally, chromosome slides were stained with Vectashield 4′,6-diamidino-2-phenylindole (DAPI) solution (Vector Laboratories, Burlingame, USA).

### Microscopy and image processing

To confirm the 2n number, karyotype structure, and FISH results, at least 20 metaphase spreads per individual were examined. Images were captured with CoolSNAP on an Axioplan II microscope (Carl Zeiss Jena GmbH, Germany) and processed with ISIS (MetaSystems Hard & Software GmbH, Altlussheim, Germany).

## Supplementary Information

Supplementary Material 1.

Supplementary Material 2.

Supplementary Material 3.

Supplementary Material 4.

Supplementary Material 5.

Supplementary Material 6.

## Data Availability

All data analyzed in this study were obtained from publicly available repositories, including NCBI GenBank and the Sequence Read Archive (SRA). The datasets comprise short-read genomic data, long-read sequencing data, complete genome assemblies, and RNA-seq data from multiple Characiformes and related species. Detailed information, including accession numbers, sequencing platforms, and sample metadata, is provided in Supplementary Table S1. Additionally, reference sequences for ribosomal RNA genes (5.8 S, 18 S, and 28 S) and the As51 satellite DNA were retrieved from GenBank under accession numbers XR_007429441.1, XR_007429016.1, XR_007429021.1, and U87962.1, respectively.

## Abbreviation

satDNAs: Satellite DNAs

## References

[1] Smith GP. Evolution of Repeated DNA Sequences by Unequal Crossover: DNA whose sequence is not maintained by selection will develop periodicities as a result of random crossover. Science. 1976;191:528–35. 10.1126/science.1251186. 1251186 10.1126/science.1251186

[2] Dover GA. Molecular drive in multigene families: How biological novelties arise, spread and are assimilated. Trends Genet. 1986;2:159–65. 10.1016/0168-9525(86)90211-8.

[3] Plohl M, Meštrović N, Mravinac B. Satellite DNA Evolution. In: Garrido-Ramos MA,Genome Dynamics., Karger S. AG; 2012. pp. 126–52. 10.1159/000337122. 10.1159/000337122 22759817

[4] Garrido-Ramos M, Satellite DNA. An Evolving Topic. Genes. 2017;8:230. 10.3390/genes8090230. 28926993 10.3390/genes8090230 PMC5615363

[5] Lower SS, McGurk MP, Clark AG, Barbash DA. Satellite DNA evolution: old ideas, new approaches. Curr Opin Genet Dev. 2018;49:70–8. 10.1016/j.gde.2018.03.003. 29579574 10.1016/j.gde.2018.03.003 PMC5975084

[6] Dos Santos RZ, Calegari RM, Silva DMZDA, Ruiz-Ruano FJ, Melo S, Oliveira C, et al. A long-term conserved satellite DNA that remains unexpanded in several genomes of Characiformes fish is actively transcribed. Genome Biol Evol. 2021;evab002. 10.1093/gbe/evab002. 10.1093/gbe/evab002 PMC8210747 33502491

[7] Oliveira C, Avelino GS, Abe KT, Mariguela TC, Benine RC, Ortí G, et al. Phylogenetic relationships within the speciose family Characidae (Teleostei: Ostariophysi: Characiformes) based on multilocus analysis and extensive ingroup sampling. BMC Evol Biol. 2011;11:275. 10.1186/1471-2148-11-275. 21943181 10.1186/1471-2148-11-275 PMC3190395

[8] Silva DMZDA, Utsunomia R, Ruiz-Ruano FJ, Daniel SN, Porto-Foresti F, Hashimoto DT, et al. High-throughput analysis unveils a highly shared satellite DNA library among three species of fish genus Astyanax. Sci Rep. 2017;7:12726. 10.1038/s41598-017-12939-7. 29018237 10.1038/s41598-017-12939-7 PMC5635008

[9] Goes CAG, Dos Santos RZ, Aguiar WRC, Alves DCV, Silva DMZDA, Foresti F, et al. Revealing the Satellite DNA History in Psalidodon and Astyanax Characid Fish by Comparative Satellitomics. Front Genet. 2022;13:884072. 10.3389/fgene.2022.884072. 35801083 10.3389/fgene.2022.884072 PMC9253505

[10] Mestriner CA, Galetti PM, Valentini SR, Ruiz IRG, Abel LDS, Moreira-Filho O, et al. Structural and functional evidence that a B chromosome in the characid fish Astyanax scabripinnis is an isochromosome. Heredity. 2000;85:1–9. 10.1046/j.1365-2540.2000.00702.x. 10971685 10.1046/j.1365-2540.2000.00702.x

[11] Abel LDDS, Mantovani M, Moreira-Filho O. Chromosomal distribution of the As51 satellite DNA in two species complexes of the genus Astyanax (Pisces, Characidae). Genet Mol Biol. 2006;29:448–52. 10.1590/S1415-47572006000300008.

[12] Kavalco KF, De Almeida-Toledo LF. Molecular Cytogenetics of Blind Mexican Tetra and Comments on the Karyotypic Characteristics of Genus *Astyanax* (Teleostei, Characidae). Zebrafish. 2007;4:103–11. 10.1089/zeb.2007.0504. 18041928 10.1089/zeb.2007.0504

[13] Kantek DLZ, Vicari MR, Peres WAM, Cestari MM, Artoni RF, Bertollo LAC, et al. Chromosomal location and distribution of As51 satellite DNA in five species of the genus *Astyanax* (Teleostei, Characidae, *Incertae sedis*). J Fish Biol. 2009;75:408–21. 10.1111/j.1095-8649.2009.02333.x. 20738546 10.1111/j.1095-8649.2009.02333.x

[14] Silva DMZA, Utsunomia R, Pansonato-Alves JC, Oliveira C, Foresti F. Chromosomal Mapping of Repetitive DNA Sequences in Five Species of *Astyanax* (Characiformes, Characidae) Reveals Independent Location of U1 and U2 snRNA Sites and Association of U1 snRNA and 5S rDNA. Cytogenet Genome Res. 2015;146:144–52. 10.1159/000438813. 26329975 10.1159/000438813

[15] Vicari MR, Artoni RF, Moreira-Filho O, Bertollo LAC. Colocalization of repetitive DNAs and silencing of major rRNA genes. A case report of the fish *Astyanax janeiroensis*. Cytogenet Genome Res. 2008;122:67–72. 10.1159/000151318. 18931488 10.1159/000151318

[16] Arnheim N, Krystal M, Schmickel R, Wilson G, Ryder O, Zimmer E. Molecular evidence for genetic exchanges among ribosomal genes on nonhomologous chromosomes in man and apes. Proc Natl Acad Sci USA. 1980;77:7323–7. 10.1073/pnas.77.12.7323. 6261251 10.1073/pnas.77.12.7323 PMC350495

[17] McStay B. Nucleolar organizer regions: genomic ‘dark matter’ requiring illumination. Genes Dev. 2016;30:1598–610. 10.1101/gad.283838.116. 27474438 10.1101/gad.283838.116 PMC4973289

[18] Zhukova A, Zakharov G, Pavlova O, Saifitdinova A. Description of the complete rDNA repeat unit structure of Coturnix japonica Temminck et Schlegel, 1849 (Aves). CCG. 2024;18:183–98. 10.3897/compcytogen.18.127373. 10.3897/compcytogen.18.127373 PMC11447458 39363903

[19] Heitkam T, Weber B, Walter I, Liedtke S, Ost C, Schmidt T. Satellite DNA landscapes after allotetraploidization of quinoa (*Chenopodium quinoa*) reveal unique A and B subgenomes. Plant J. 2020;103:32–52. 10.1111/tpj.14705. 31981259 10.1111/tpj.14705

[20] Garcia S, Pascual-Díaz JP, Krumpolcová A, Kovarík A. Analysis of 5S rDNA Genomic Organization Through the RepeatExplorer2 Pipeline: A Simplified Protocol. In: Heitkam T, Garcia S, editors. Plant Cytogenetics and Cytogenomics. New York, NY: Springer US; 2023. pp. 501–12. 10.1007/978-1-0716-3226-0_30. 10.1007/978-1-0716-3226-0_30 37335496

[21] Vondrak T, Ávila Robledillo L, Novák P, Koblížková A, Neumann P, Macas J. Characterization of repeat arrays in ultra-long nanopore reads reveals frequent origin of satellite DNA from retrotransposon‐derived tandem repeats. Plant J. 2020;101:484–500. 10.1111/tpj.14546. 31559657 10.1111/tpj.14546 PMC7004042

[22] Mann L, Balasch K, Schmidt N, Heitkam T. High-fidelity (repeat) consensus sequences from short reads using combined read clustering and assembly. BMC Genomics. 2024;25:109. 10.1186/s12864-023-09948-4. 38267856 10.1186/s12864-023-09948-4 PMC10809544

[23] Elphinstone C, Elphinstone R, Todesco M, Rieseberg LH, RepeatOBserver. Tandem Repeat Visualisation and Putative Centromere Detection. Mol Ecol Resour. 2025;25:e14084. 10.1111/1755-0998.14084. 40035343 10.1111/1755-0998.14084 PMC12415947

[24] Dyomin A, Galkina S, Fillon V, Cauet S, Lopez-Roques C, Rodde N, et al. Structure of the intergenic spacers in chicken ribosomal DNA. Genet Sel Evol. 2019;51:59. 10.1186/s12711-019-0501-7. 31655542 10.1186/s12711-019-0501-7 PMC6815422

[25] Davidian AG, Dyomin AG, Galkina SA, Makarova NE, Dmitriev SE, Gaginskaya ER. 45S rDNA Repeats of Turtles and Crocodiles Harbor a Functional 5S rRNA Gene Specifically Expressed in Oocytes. Mol Biol Evol. 2022;39:msab324. 10.1093/molbev/msab324. 34905062 10.1093/molbev/msab324 PMC8789306

[26] Bendich AJ, Rogers SO. Ribosomal Intergenic Spacers Are Filled with Transposon Remnants. Genome Biol Evol. 2023;15:evad114. 10.1093/gbe/evad114. 37341531 10.1093/gbe/evad114 PMC10319768

[27] Charlesworth B, Sniegowski P, Stephan W. The evolutionary dynamics of repetitive DNA in eukaryotes. Nature. 1994;371:215–20. 10.1038/371215a0. 8078581 10.1038/371215a0

[28] Jo S-H, Koo D-H, Kim JF, Hur C-G, Lee S, Yang T, et al. Evolution of ribosomal DNA-derived satellite repeat in tomato genome. BMC Plant Biol. 2009;9:42. 10.1186/1471-2229-9-42. 19351415 10.1186/1471-2229-9-42 PMC2679016

[29] Jo S-H, Park H-M, Kim S-M, Kim HH, Hur C-G, Choi D. Unraveling the sequence dynamics of the formation of genus-specific satellite DNAs in the family solanaceae. Heredity. 2011;106:876–85. 10.1038/hdy.2010.131. 21063436 10.1038/hdy.2010.131 PMC3186232

[30] Sviggum SM, Goes CAG, Dziechciarz Vidal JA, Dos Santos RZ, Gisloti-Ribeiro ME, Desajacomo LM, et al. Comparative analysis reveals highly conserved satellite DNA landscapes in two sympatric *Gymnotus* (Teleostei, Gymnotiformes) electric knifefish. Genome. 2025;68:1–10. 10.1139/gen-2025-0043. 41100897 10.1139/gen-2025-0043

[31] Ruiz-Ruano FJ, López-León MD, Cabrero J, Camacho JPM. High-throughput analysis of the satellitome illuminates satellite DNA evolution. Sci Rep. 2016;6:28333. 10.1038/srep28333. 27385065 10.1038/srep28333 PMC4935994

[32] Valeri MP, Dias GB, Pereira VDS, Campos Silva Kuhn G, Svartman M. An eutherian intronic sequence gave rise to a major satellite DNA in Platyrrhini. Biol Lett. 2018;14:20170686. 10.1098/rsbl.2017.0686. 29386361 10.1098/rsbl.2017.0686 PMC5803596

[33] Belyayev A, Josefiová J, Jandová M, Mahelka V, Krak K, Mandák B. Transposons and satellite DNA: on the origin of the major satellite DNA family in the Chenopodium genome. Mob DNA. 2020;11:20. 10.1186/s13100-020-00219-7. 32607133 10.1186/s13100-020-00219-7 PMC7320549

[34] Oliveira C, Biodiversity. Systematics, and Taxonomy of Ostariophysi (Osteichthyes, Actinopterygii): What We Know Today After Three Decades of Integration of Morphological and Molecular Data. Taxonomy. 2025;5:33. 10.3390/taxonomy5020033.

[35] Melo BF, Ota RP, Benine RC, Carvalho FR, Lima FCT, Mattox GMT, et al. Phylogenomics of Characidae, a hyper-diverse Neotropical freshwater fish lineage, with a phylogenetic classification including four families (Teleostei: Characiformes). Zool J Linn Soc. 2024;202:zlae101. 10.1093/zoolinnean/zlae101.

[36] Medrado AS, De Mello Affonso PRA, Carneiro PLS, Vicari MR, Artoni RF, Costa MA. Allopatric divergence in Astyanax aff. fasciatus Cuvier, 1819 (Characidae, Incertae sedis) inferred from DNA mapping and chromosomes. Zoologischer Anzeiger - J Comp Zool. 2015;257:119–29. 10.1016/j.jcz.2015.05.005.

[37] Altschul SF, Gish W, Miller W, Myers EW, Lipman DJ. Basic local alignment search tool. J Mol Biol. 1990;215:403–10. 10.1016/S0022-2836(05)80360-2. 2231712 10.1016/S0022-2836(05)80360-2

[38] Langmead B, Salzberg SL. Fast gapped-read alignment with Bowtie 2. Nat Methods. 2012;9:357–9. 10.1038/nmeth.1923. 22388286 10.1038/nmeth.1923 PMC3322381

[39] Edgar RC. MUSCLE: multiple sequence alignment with high accuracy and high throughput. Nucleic Acids Res. 2004;32:1792–7. 10.1093/nar/gkh340. 15034147 10.1093/nar/gkh340 PMC390337

[40] Seibt KM, Schmidt T, Heitkam T. FlexiDot: highly customizable, ambiguity-aware dotplots for visual sequence analyses. Bioinformatics. 2018;34:3575–7. 10.1093/bioinformatics/bty395. 29762645 10.1093/bioinformatics/bty395

[41] Cioffi MB, Martins C, Centofante L, Jacobina U, Bertollo LAC. Chromosomal Variability among Allopatric Populations of Erythrinidae Fish *Hoplias malabaricus*: Mapping of Three Classes of Repetitive DNAs. Cytogenet Genome Res. 2009;125:132–41. 10.1159/000227838. 19729917 10.1159/000227838

[42] Sassi FDMC, Toma GA, De Bello Cioffi M. FISH—in Fish Chromosomes. In: Cytogenetics and Molecular Cytogenetics. 1st edition. Boca Raton: CRC Press; 2022. pp. 281–96. 10.1201/9781003223658-24.

