# Supplementary figures and images for: Evolutionary trends of As51 satellite DNA and its colocalization with 45S ribosomal DNA: a conserved feature in Characiformes fishes
Source: BMC Genomics. 2026 Apr 1;27:459. doi: 10.1186/s12864-026-12792-x (PMC13170303; doi:10.1186/s12864-026-12792-x)

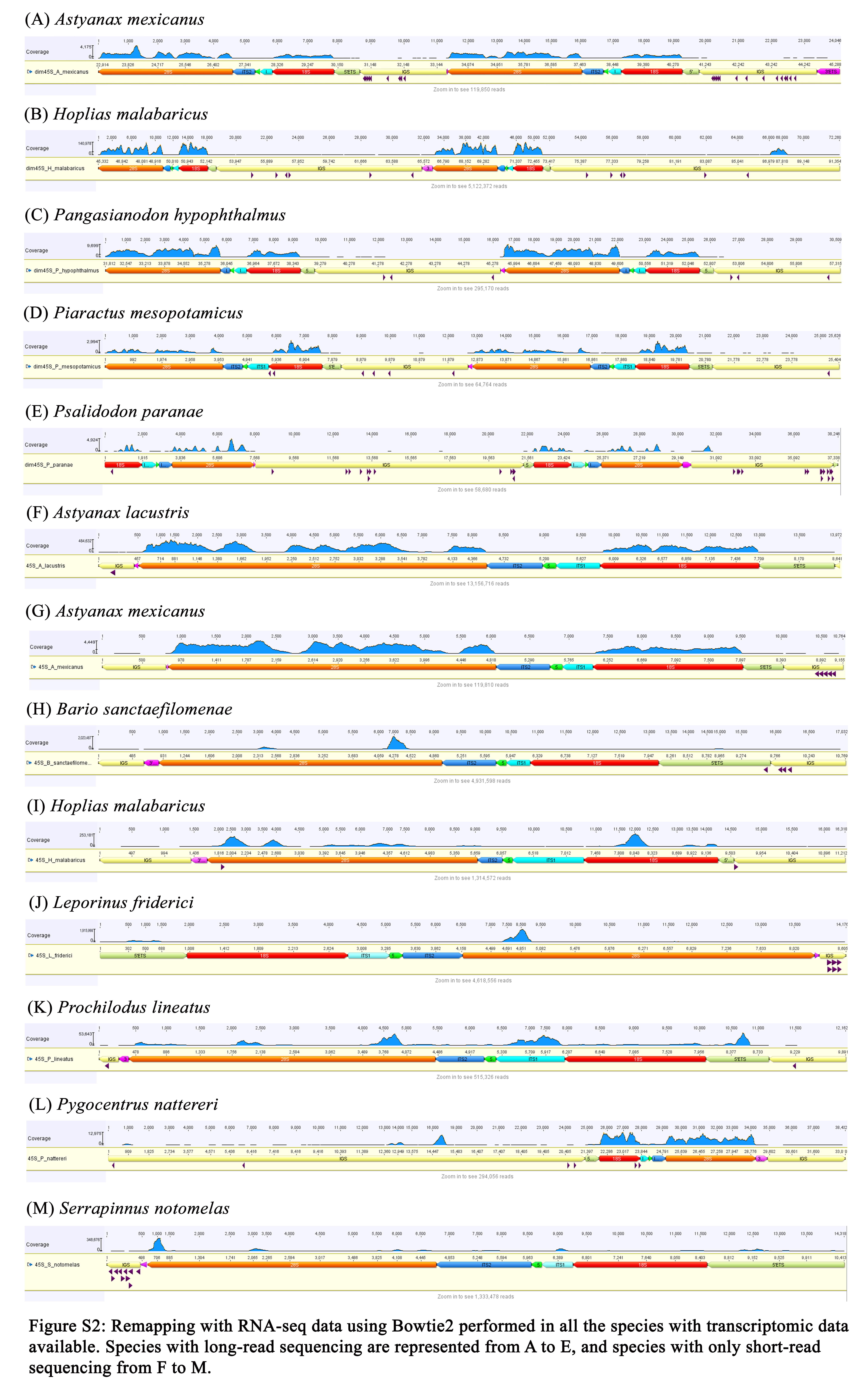

Supplement: Supplementary file 2 — Supplementary Material 2. [file 12864_2026_12792_MOESM2_ESM.jpeg]

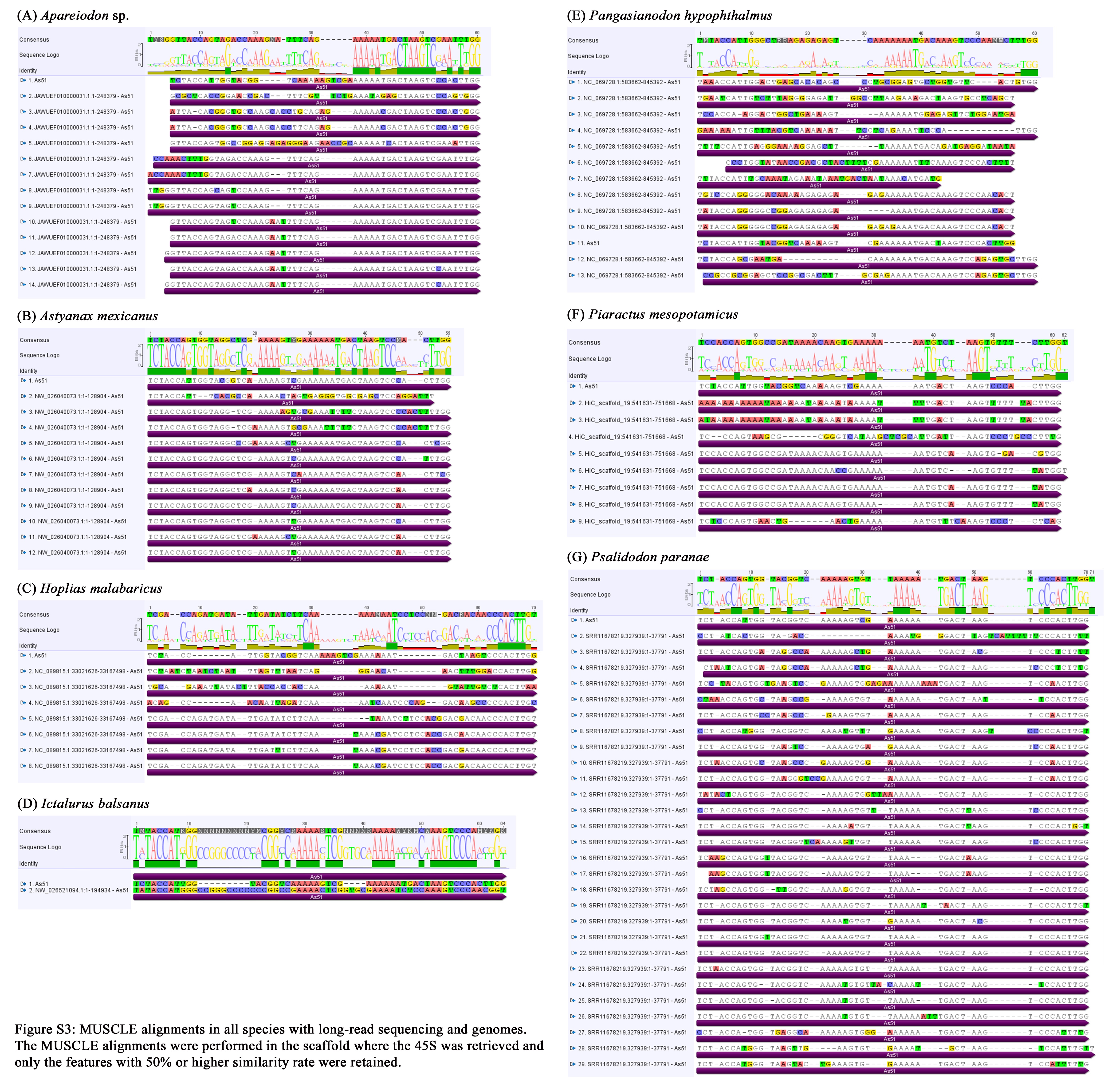

Supplement: Supplementary file 3 — Supplementary Material 3. [file 12864_2026_12792_MOESM3_ESM.jpeg]

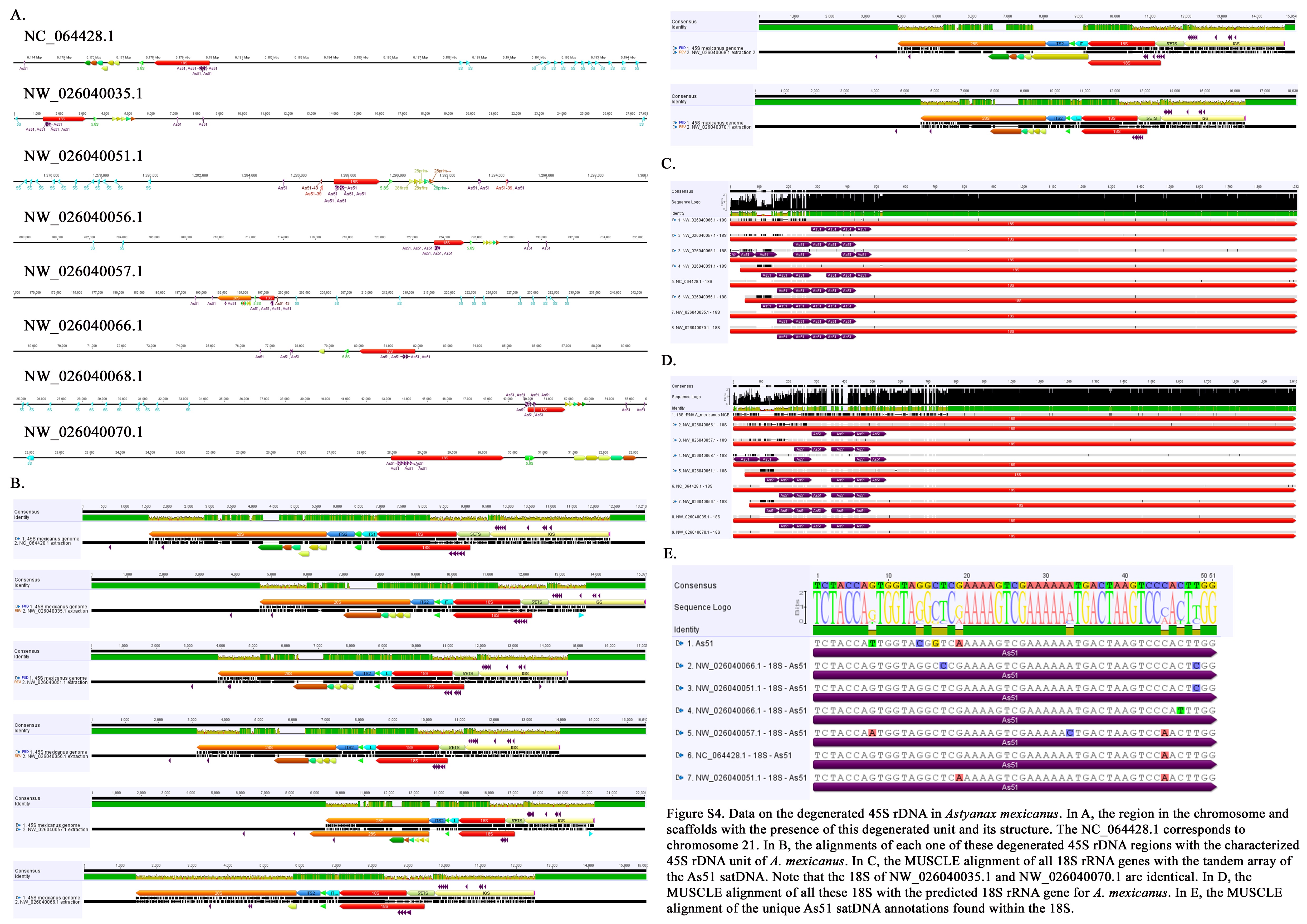

Supplement: Supplementary file 4 — Supplementary Material 4. [file 12864_2026_12792_MOESM4_ESM.jpeg]

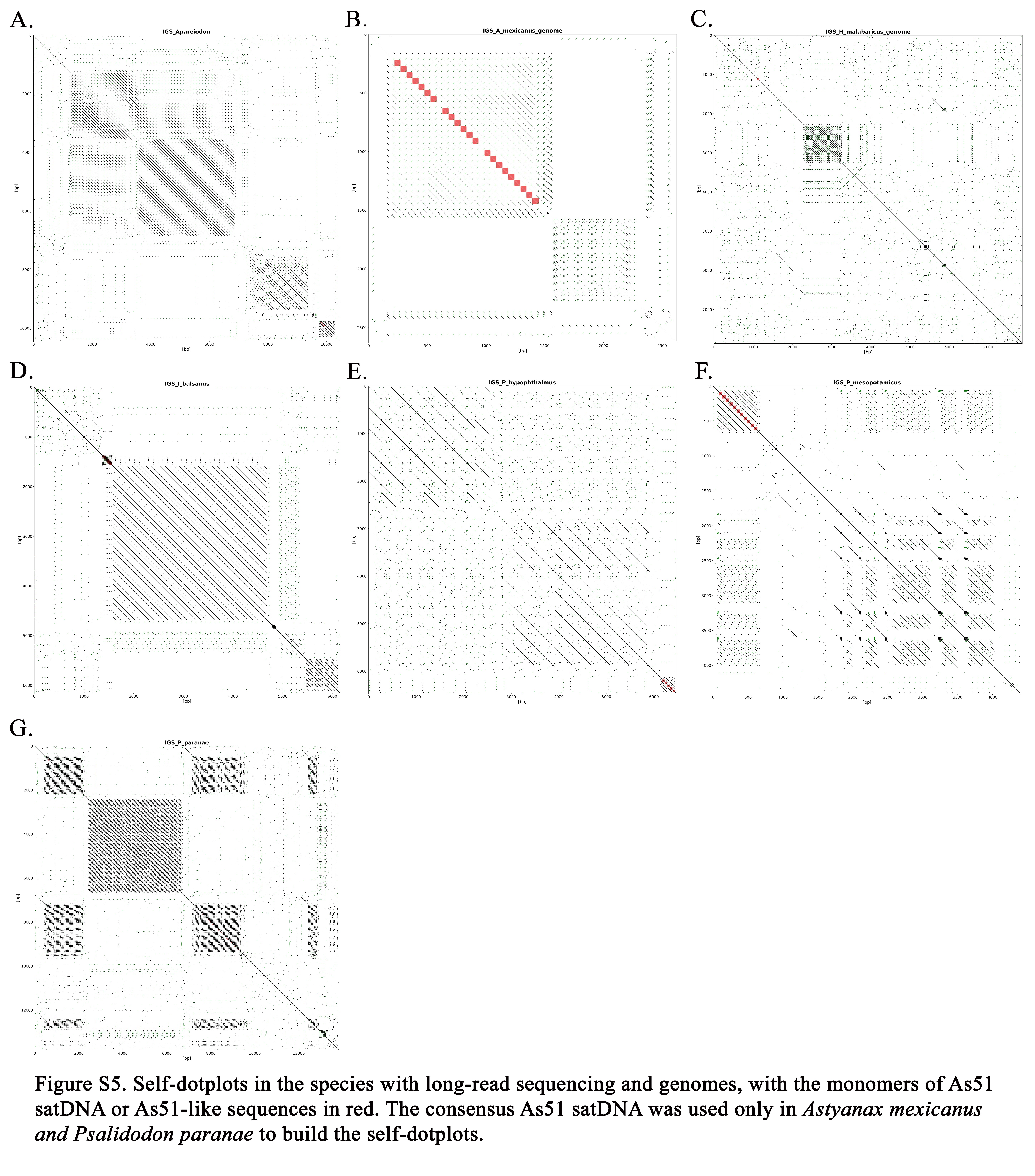

Supplement: Supplementary file 5 — Supplementary Material 5. [file 12864_2026_12792_MOESM5_ESM.jpeg]

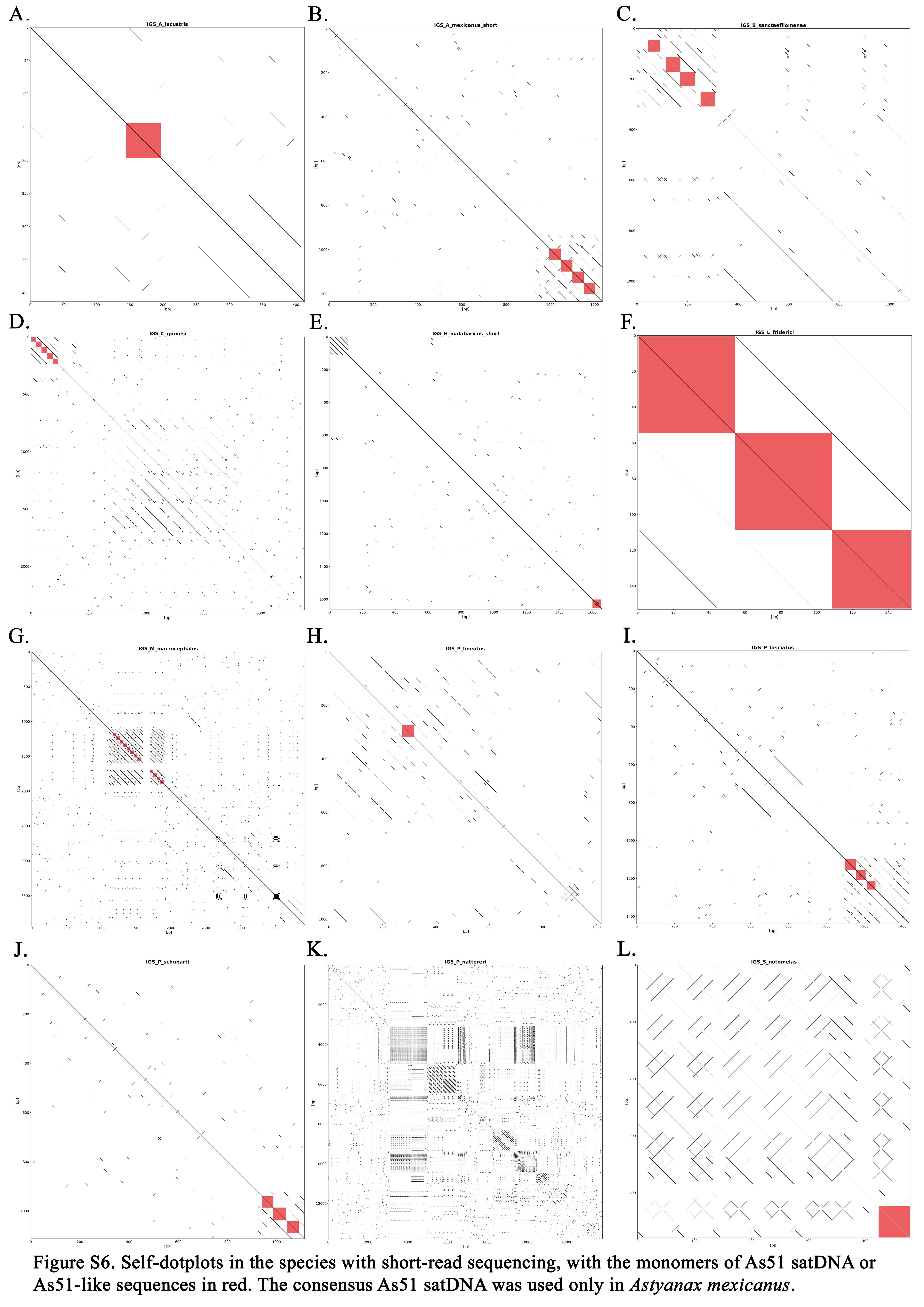

Supplement: Supplementary file 6 — Supplementary Material 6. [file 12864_2026_12792_MOESM6_ESM.jpeg]
